# P-1344. Molecular Characterization of Daptomycin Non-susceptible *Staphylococcus aureus*

**DOI:** 10.1093/ofid/ofae631.1521

**Published:** 2025-01-29

**Authors:** Geehan Suleyman, Jagjeet Kaur, Xiangmin Zhang, Robert Tibbetts, Marcus Zervos, George J Alangaden

**Affiliations:** Henry Ford Health, Detroit, Michigan; Henry Ford Health, Detroit, Michigan; Wayne State University, Detroit, Michigan; Henry Ford Health, Detroit, Michigan; Henry Ford Hospital, Detroit, Michigan; Henry Ford Health, Detroit, Michigan

## Abstract

**Background:**

*Staphylococcus aureus* (SA) is the leading cause of bacteremia in hospitalized patients. With limited therapeutic options, increasing use of daptomycin (DAP) has resulted in the emergence of DAP non-susceptible (DNS) SA strains. Nevertheless, the mechanisms of resistance in DNS SA are not fully understood. Proposed mechanisms include genetic and phenotypic changes affecting the cell membrane and cell wall. However, studies have been limited to single pairs of isolates or isolates that are laboratory derived.

**Methods:**

We performed whole-genome sequencing (WGS) on clinically obtained SA blood isolates at Henry Ford Health, a comprehensive, integrated, health care organization in Southeast Michigan from 2005-2023. DNS was defined as MIC >1. Sequencing libraries were created using QIAseq FX DNA Library Kit (Qiagen, USA) according to manufacturer’s instructions and sequenced on NovaSeq 6000 (Illumina, San Diego, USA). FastQ files were further analyzed for the presence of genetic variations using a WGS bioinformatic pipeline based on *de novo* assembly.

**Results:**

We sequenced 199 SA isolates from 59 patients, of which 88 (44%) were DNS. 43 (73%) patients had multiple isolates, whereas 16 (27%) only had one. All DNS isolates except one were MRSA. 46 (52%) DNS isolates had amino acid substitution in the MprF and 2 (2%) in the cls genes, which are known to be associated with DNS. 10 (11%) DNS isolates were found to have substitutions (S829L and L406I) in the MprF gene that have not been previously reported. Mutations in cls, rpoB, rpoC, walK and agrA genes were also detected. All isolates with known mutations were exposed to DAP prior to development of DNS. 30 (34%) DNS isolates did not have mutations associated with resistance. Of these, only 13 (43%) had prior exposure to DAP, 9 (30%) were only exposed to vancomycin, and 11 (37%) were not exposed to any antibiotics. ST5 and ST8 were the most common sequence types.

**Conclusion:**

In this study involving a large number of clinical SA isolates, the majority of patients who developed DNS due to MprF mutations were exposed to DAP, likely due to selection pressure. However, more than one-third of DNS isolates did not exhibit resistance mutations. Further studies are needed to identify other mechanisms of DAP resistance, especially in those with no prior antibiotic exposure.

**Disclosures:**

**Marcus Zervos, MD**, Johnson and johnson: Grant/Research Support|Moderna: Grant/Research Support

